# Kif18a regulates Sirt2-mediated tubulin acetylation for spindle organization during mouse oocyte meiosis

**DOI:** 10.1186/s13008-018-0042-4

**Published:** 2018-11-10

**Authors:** Feng Tang, Meng-Hao Pan, Xiang Wan, Yujie Lu, Yu Zhang, Shao-Chen Sun

**Affiliations:** College of Animal Science and Technology, Nanjing Agricultural University, Nanjing, 210095 China

**Keywords:** Kif18a, Oocyte, Meiosis, Spindle, Acetylation

## Abstract

**Background:**

During oocyte meiosis, the cytoskeleton dynamics, especially spindle organization, are critical for chromosome congression and segregation. However, the roles of the kinesin superfamily in this process are still largely unknown.

**Results:**

In the present study, Kif18a, a member of the kinesin-8 family, regulated spindle organization through its effects on tubulin acetylation in mouse oocyte meiosis. Our results showed that Kif18a is expressed and mainly localized in the spindle region. Knock down of Kif18a caused the failure of first polar body extrusion, dramatically affecting spindle organization and resulting in severe chromosome misalignment. Further analysis showed that the disruption of Kif18a caused an increase in acetylated tubulin level, which might be the reason for the spindle organization defects after Kif18a knock down in oocyte meiosis, and the decreased expression of deacetylase Sirt2 was found after Kif18a knock down. Moreover, microinjections of tubulin K40R mRNA, which could induce tubulin deacetylation, protected the oocytes from the effects of Kif18a downregulation, resulting in normal spindle morphology in Kif18a-knock down oocytes.

**Conclusions:**

Taken together, our results showed that Kif18a affected Sirt2-mediated tubulin acetylation level for spindle organization during mouse oocyte meiosis. Our results not only revealed the critical effect of Kif18a on microtubule stability, but also extended our understanding of kinesin activity in meiosis.

## Background

During mammalian oocyte maturation, the cytoskeleton, which is mainly constructed of microtubules and microfilaments, guarantees accurate chromosome congression, segregation and meiotic cell division [3, 20]. Microtubules are hollow cylindrical tubes consisting of 13 aligned protofilaments composed of α-tubulin and β-tubulin [27, 34]. Free tubulins in the cytoplasm assemble and organize to form a meiotic spindle, which is involved in chromosome alignment and correct chromosome segregation during oocyte meiosis. During this process, post-translational modifications of tubulin (including acetylation, tyrosination and polyglutamylation) are necessary for microtubule-based functions [31]. Microtubule stability depends on the acetylation of tubulin, and this modification normally occurs in the lysine 40 residue of tubulin [13, 28].

The kinesin superfamily, which was first discovered in the brains of squid and mammals [36], has been reported to participate in a series of vital cellular processes. As motor proteins, kinesins are thought to fulfill two main functions. The first is that kinesins hydrolyze ATP to provide energy which is used to transport substances that combine with the kinesin cargo domain. For example, Kinesin-2 plays an essential role in the intraflagellar-transport machinery [24]. The other is that kinesins are involved in spindle assembly and chromosome alignment in mitotic cells. For instance, it is reported that Kinesin-5 affects kinetochore-microtubule attachment, facilitating chromosome congression in *Drosophila* melanogaster S2 cells [35]. Additionally, Kif4 is proved to control microtubule stabilization and cell migration through the Rho-mDia-EB1 pathway in the lamella of fibroblasts [25].

Kif18a is one of the 45 kinesin proteins discovered in mouse and is a member of the kinesin-8 family together with Kif18b [22]. The Kif18 subfamily has been revealed to possess core functions related to cell development in different species. Several studies have shown that Kif18a is a novel biomarker for breast and colorectal cancer [26, 40], and Kif18a overexpression is also associated with unfavorable prognosis in primary hepatocellular carcinoma [16, 19]. Recent studies indicated that Kif18b contributes to spindle positioning through regulation of astral microtubule length [33], whereas the absence of Kif18b inhibits centrosome separation in Kif18b knock out mice [37]. Similarly to Kif18b, Kif18a is shown to regulate kinetochore-microtubule attachment dynamics, affecting chromosome positioning during mitosis [7]. It is also reported that Kif18a attenuates centromere movement via its effect on microtubule pausing [32]. Moreover, KIp67A in *Drosophila*, an orthologue of the human Kif18a, controls chromosome alignment and spindle length through interaction with kinetochores [29]. In mammalian male meiosis, Kif18a impairs chromosome congression and dysregulates BubR1 and CENP-E, however, Kif18a knockout females are fertile, and Kif18a knock out ovaries exhibited no apparent histological defects [17]. While in *Drosophila*, the kinesin-like protein KLP67A is essential for mitotic and male meiotic spindle assembly [12]. However, the role of Kif18a in mammalian female meiosis is still poorly understood.

In this study, we employed the knock-down approach to study the functions of Kif18a in mammalian oocyte meiosis. We hypothesized that Kif18 expressed in oocytes and regulated spindle in oocytes. And our results confirmed that Kif18a is localized at the spindle and affects microtubule stability for spindle organization during mouse oocyte meiosis. This study offers an insight into the roles of Kif18a during tubulin acetylation in mammalian germ cells.

## Results

### Expression and localization of Kif18a in mouse oocytes

**Fig. 1:**
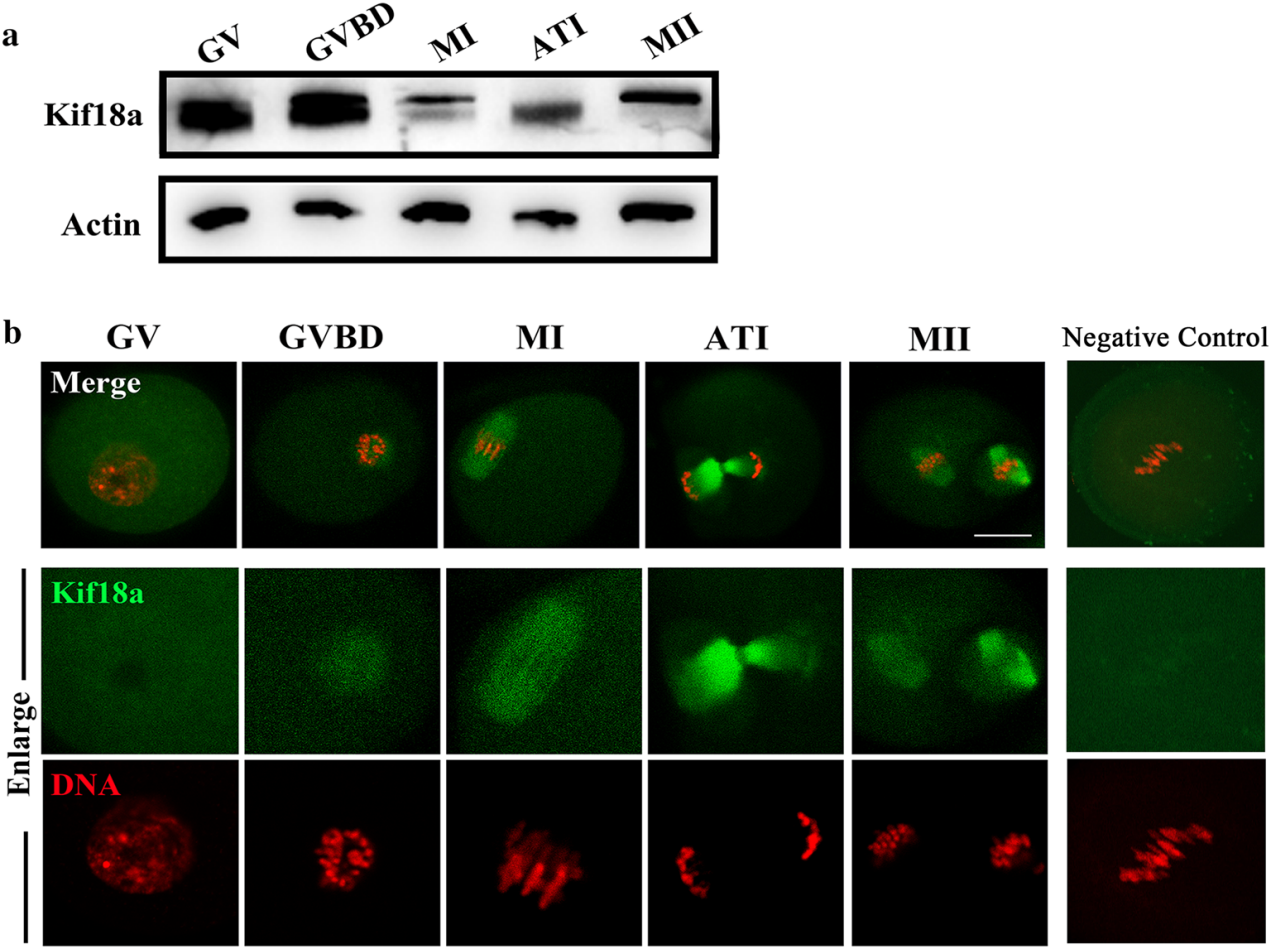
Expression and localization of Kif18a in mouse oocytes. **a** Oocytes at different maturation stages were examined by Western blotting. **b** Oocytes from GV to MII stages were stained with anti-Kif18a antibody (green) and counterstained with DAPI for DNA visualization (red). After GVBD, Kif18a accumulated around chromosomes. Also, Kif18a localized in the meiotic spindle region at both the MI and MII stages, while it localized in the midbody at the ATI stage. Negative control was stained with secondary antibody without Kif18a. Scale bar: 20 μm

We first examined whether Kif18a is expressed in oocytes. The different stages of oocytes were examined by western blotting, which revealed that Kif18a is expressed at all stages during oocyte maturation (Fig. 1a). Subsequently, we examined the subcellular localization pattern of Kif18a in mouse oocytes. As shown in Fig. 1b, Kif18a had no specific localization pattern in the germ vesicle (GV) stage, whereas, after germ vesicle break down (GVBD), Kif18a accumulated around the chromosomes; in the metaphase I (MI) and metaphase II (MII) stages, Kif18a was enriched in the meiotic spindle region, and Kif18a localized in the middle body during the anaphase-telophase I (ATI) stage. The localization pattern of Kif18a indicates a relationship between Kif18a and the spindle region during mouse oocyte meiosis.

### The localization of Kif18a changes after taxol and nocodazole treatment

**Fig. 2:**
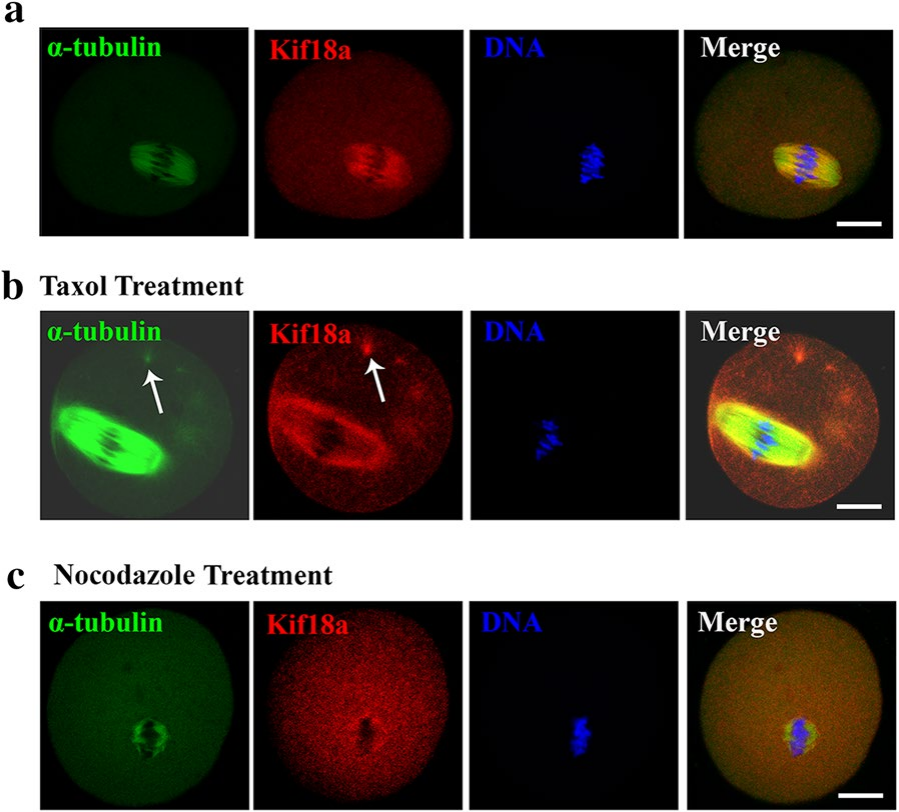
Kif18 localization after taxol or nocodazole treatment. **a** Double staining of MI oocytes with an anti-Kif18a antibody (red) and an anti-α-tubulin antibody (green). Oocytes were counterstained with DAPI to visualize DNA (blue). Kif18a mainly localized on the meiotic spindle. **b** Subcellular localization of Kif18a after taxol treatment during mouse oocyte meiotic maturation. Arrows indicated asters. Green, α-tubulin; red, Kif18a. **c** Subcellular localization of Kif18a after nocodazole treatment during mouse oocyte meiotic maturation. Green, α-tubulin; red, Kif18a. Scale bar: 20 μm

To confirm the localization pattern of Kif18a, oocytes were double-stained using the anti-Kif18a and anti-α-tubulin antibodies, and results showed that Kif18a co-localizes with microtubules in oocytes (Fig. 2a). Subsequently, we disturbed the microtubules to determine whether the localization of Kif18a would also change. After taxol treatment, the microtubules were stabilized and asters appeared in the cytoplasm; Kif18a localized at the asters in these oocytes (Fig. 2b). In contrast, after nocodazole treatment, the microtubules were depolymerized and there was no spindle formation. In this case, Kif18a also showed no specific localization and dispersed in the cytoplasm (Fig. 2c). These results indicate that the localization pattern of Kif18a changed after microtubule drug treatment in mouse oocytes.

### Depletion of Kif18a affects polar body extrusion in mouse oocytes

**Fig. 3:**
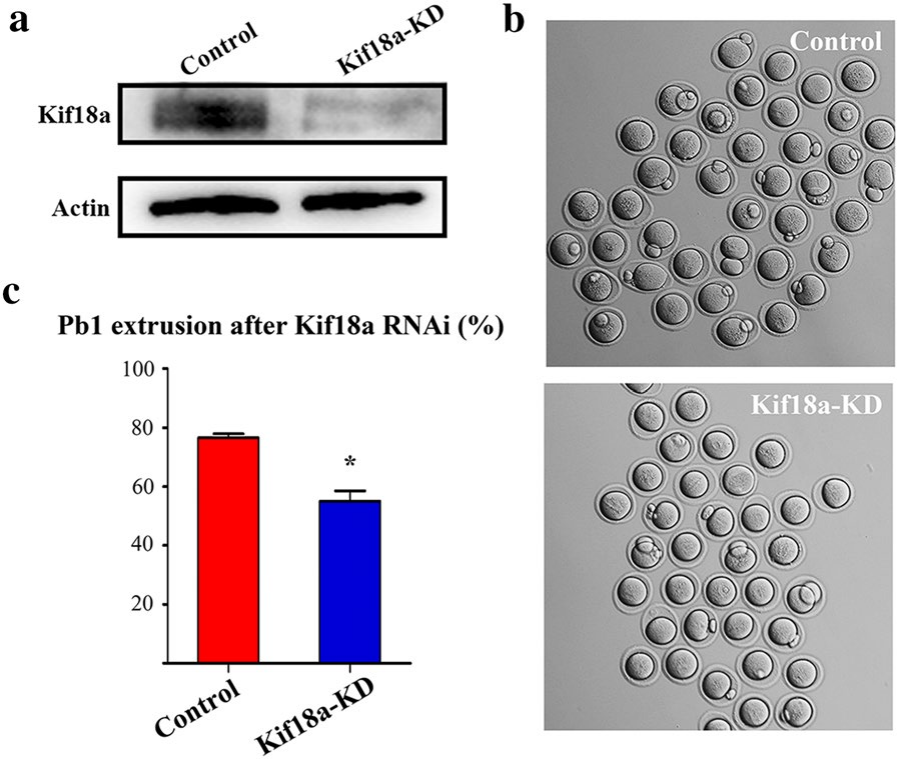
Knockdown of Kif18a affects mouse oocyte maturation. **a** Western blotting was employed to detect the endogenous Kif18a protein expression after knockdown of Kif18a. Three independent experiments were performed. **b** Typical phase-contrast image of control siRNA-injected and Kif18a-KD oocytes. **c** The rate of first polar body extrusion in control and Kif18-KD oocytes. Data are presented as mean percentage (± SEM) of at least three independent experiments. *p < 0.05

To explore the potential roles of Kif18a in meiosis, we employed siRNA microinjection to knock down Kif18a protein expression. Obtained results showed that Kif18a protein expression was effectively reduced after Kif18a siRNA microinjection (Fig. 3a). Subsequently, a part proportion of treated oocytes failed to extrude first polar body (Fig. 3b), and statistical analysis suggested that the rate of first polar body extrusion was dramatically lower in the Kif18a-KD oocytes than in the control oocytes (55.0 ± 3.61%, n = 218 vs. 76.67 ± 1.33%, n = 175, p < 0.05; Fig. 3c). These results indicate that Kif18a is essential for mouse oocyte maturation.

### Kif18a regulates the α-tubulin acetylation level for spindle organization

**Fig. 4:**
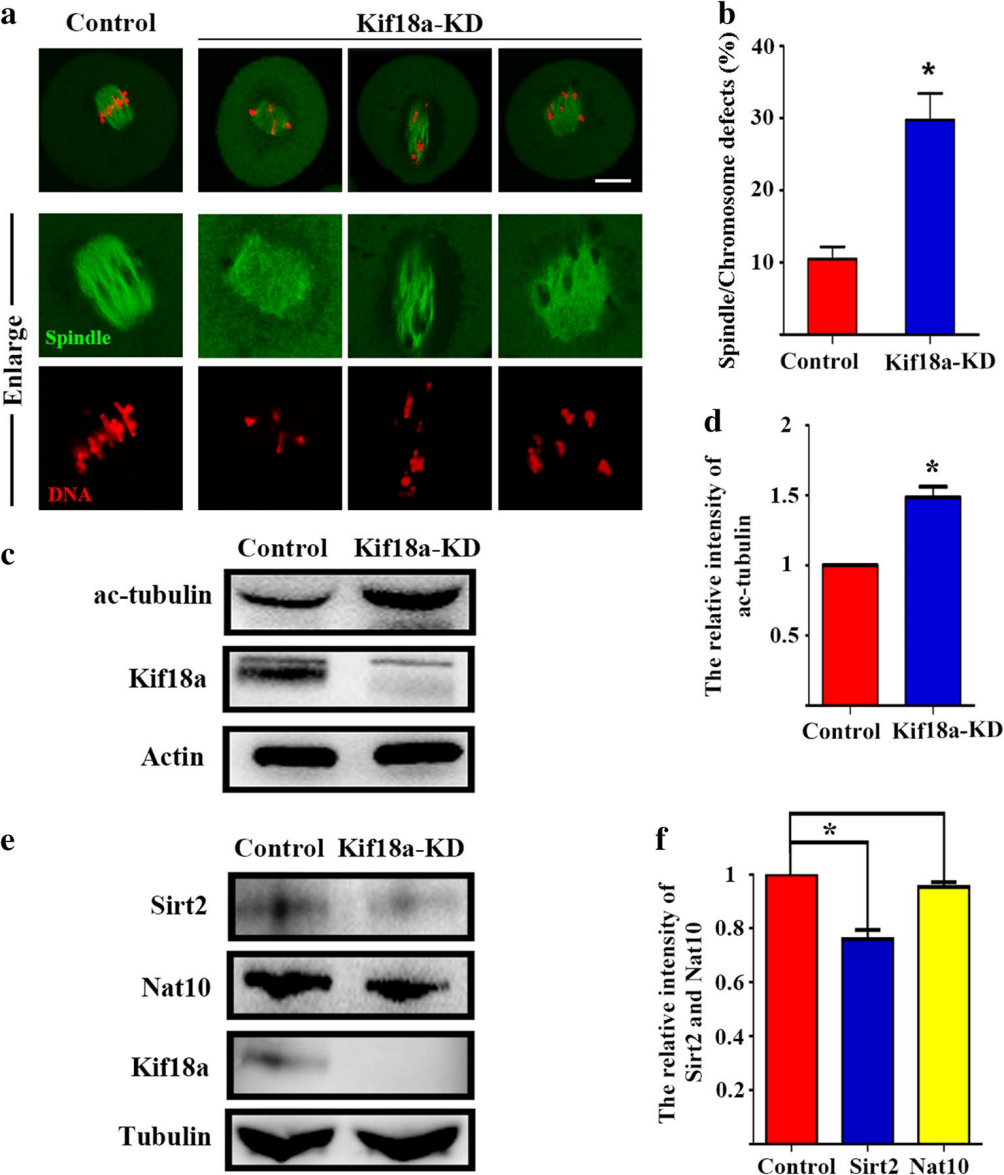
Knockdown of Kif18a leads to spindle and chromosomal defects during meiosis. **a** Typical images of MI oocytes after microinjection of control siRNA and Kif18a siRNA. Spindles of control MI oocytes exhibited the classical barrel shape with organized chromosomes, while malformed and multipolar spindles occurred in Kif18a-KD oocytes. Green, α-tubulin; red, DNA. **b** The incidence of abnormal spindles and misaligned chromosomes in control and Kif18a-KD oocytes. **c** Endogenous ac-tubulin protein expression in control and Kif18a-KD oocytes was examined by Western blotting. **d** Relative intensities of the Kif18a protein bands in control and Kif18a-KD oocytes. Ac-tubulin protein expression was significantly increased in Kif18a-KD oocytes. **e** Sirt2 and Nat10 expression in control and Kif18a-KD oocytes was examined by Western blotting. After Kif18a knock down, the expression of Sirt2 decreased while there was no difference for Nat10. **f** Relative intensities of Sirt2 and Nat10 protein bands in control and Kif18a-KD oocytes. Data are presented as mean percentage (± SEM) of at least three independent experiments. *p < 0.05; scale bars: 20 μm

Next, we attempted to determine the reasons for the oocyte maturation defects caused by the Kif18a knockdown (KD). Due to the co-localization of Kif18a and α-tubulin, spindle morphology was examined. The spindles of Kif18a-KD oocytes exhibited severe morphological defects (malformed and multipolar spindles), while the spindles of control oocytes presented the classical barrel shape with organized chromosomes (Fig. 4a). Moreover, the chromosome alignment was also disrupted by the Kif18a KD. The incidence of spindle defects was significantly higher in Kif18a-KD oocytes than in control oocytes (29.77 ± 3.66%, n = 91 vs. 10.47 ± 1.70%, n = 85, p < 0.05; Fig. 4b). To determine the mechanism underlying the effects of Kif18a on spindle organization, we examined the ac-tubulin level, since the posttranslational modifications of microtubules, such as acetylation, tyrosination, and polyglutamylation, are essential for spindle biological functions. After Kif18a KD, ac-tubulin protein expression was significantly higher in the Kif18a-KD oocytes than the control oocytes (Fig. 4c). This was also confirmed by the calculation of the relative densities of the protein bands (1 vs 1.48 ± 0.06, p < 0.05; Fig. 4d). To find the link for the Kif18 and tubulin acetylation, we examined the protein expression of deacetylase Sirt2 and acetyltransferase Nat10, and our results showed that the expression of Sirt2 was decreased after Kif18a knock down, while there was no difference for the expression of Nat10 (Fig. 4e). Band intensity analysis also confirmed this (Sirt2: 1 vs 0.76 ± 0.028; p < 0.05; Nat10: 1 vs 0.95 ± 0.014; p > 0.05; Fig. 4f). Our results suggest that Kif18a affects tubulin acetylation, impacting meiotic spindle formation in mouse oocytes.

### Microinjection of tubulin K40R mRNA rescues mouse oocytes from Kif18a deficiency

**Fig. 5:**
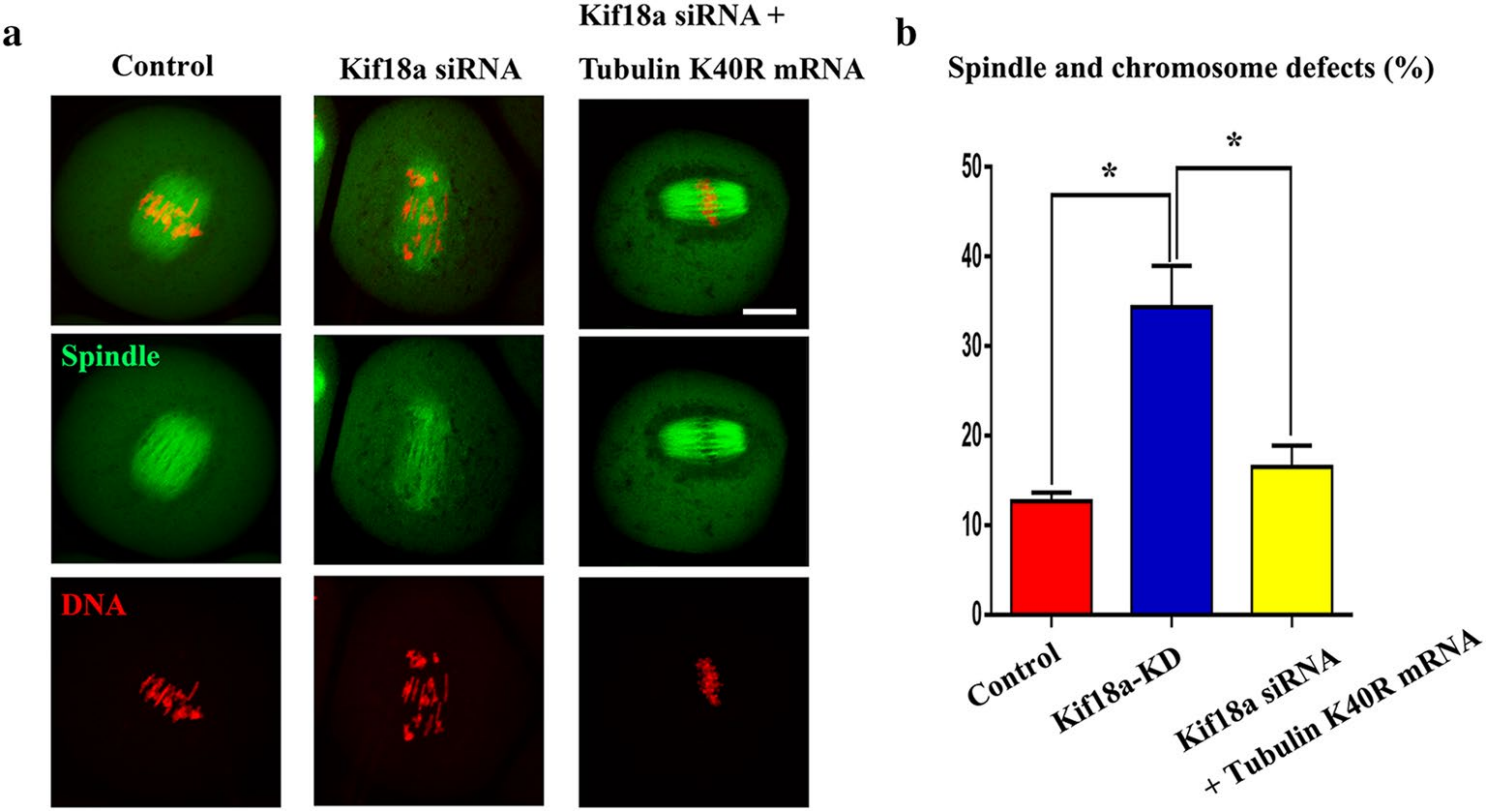
Spindle assembly and chromosome alignment demand hypoacetylation of tubulin K40 during meiosis. **a** Typical images of MI control oocytes, Kif18a siRNA oocytes and Kif18a siRNA + tubulin K40R mRNA-injected oocytes. Green, α-tubulin; red, DNA. **b** The incidence of abnormal spindles and misaligned chromosomes in control oocytes, Kif18a siRNA oocytes and Kif18a siRNA + tubulin K40R mRNA-injected oocytes. Data are presented as mean percentage (± SEM) of at least three independent experiments. *p < 0.05; scale bars: 20 μm

Tubulin acetylation normally occurs at lysine 40, and the substitution of lysine (K) with arginine (R) can imitate deacetylation. Therefore, we investigated whether tubulin K40R could revitalize abnormal oocytes with spindle and chromosome defects incurred due to the high acetylation level of tubulin resulting from Kif18a KD. For this purpose, tubulin K40R mRNA was injected into Kif18a-deficient oocytes. We examined spindle morphology and found that the microinjection of tubulin K40R mRNA significantly reduced the proportion of abnormal spindle/chromosomes in Kif18a-KD oocytes (Fig. 5a), which was confirmed by statistical analysis (34.37 ± 4.62%, n = 86 vs 16.47 ± 2.43%, n = 88 rescue vs 12.63 ± 0.99%, n = 109 control, Fig. 5b).

## Discussion

In the present study, Kif18a was found to be expressed in mouse oocytes, being closely associated with microtubules. Furthermore, the depletion of Kif18a induced oocyte maturation defects, which may be due to its effects on tubulin acetylation-related spindle organization.

The kinesin superfamily proteins are associated with microtubules and are involved in a series of cellular activities related to spindle organization and chromosome movement [11]. For example, Kif25 establishes proper spindle orientation via inhibition of centrosome separation [8]. Meanwhile, both Kif4 and Kif17 were found to be indispensable for microtubule stabilization [1, 25]. In female meiosis, only a few kinesins were studied, and these kinesins were respectively related with cell progression, spindle formation, and chromosome separation in meiosis [5]. In the present study, we showed that Kif18a is expressed in mouse oocytes during meiosis, being closely associated with microtubules. Furthermore, the knockdown of Kif18a induced oocyte maturation defects, possibly through its effects on spindle organization. Studies in other models also provide evidence for the effect of Kif18a on spindle formation. The Kif18 subfamily contains two members (Kif18a, Kif18b) [22]. The Kif18b gene is localized on chromosome 11 and is encoded by 17 exons, presenting functions like the regulation of spatial microtubule organization in PtK cells and modulation of spindle formation in EIC cells by the eg5-dependent pathway [21, 37]. As a counterpart of Kif18b, the Kif18a gene is localized on chromosome 2 and is also encoded by 17 exons. Kif18a is required for mitotic progression during germ line development [7]. Furthermore, in Hela cells, proper chromosome alignment dependents on the negative manipulation of kinetochore oscillation based on Kif18a [17]. Also, Kif18a was found to affect polymerization dynamics of microtubule plus ends without destabilizing them in budding yeast [10]. Moreover, in *Xenopus laevis* Kif18a was shown to regulate meiotic spindle integrity [23]. Consistent with these previous studies, our results using a mouse oocyte model indicated the conserved role of Kif18a on microtubules in different species and models.

To determine the potential mechanism for the role of Kif18a on spindle organization, we examined tubulin acetylation (ac-tubulin) level. Acetylation of tubulin widely impacts a series of cellular activities. Alpha-tubulin was first found to be post-translationally modified by acetylation on the epsilon-amino group of a lysine, and as a modified protein existing in primitive protist *Trichomonas vaginalis* [9, 14]. Further investigation showed that ac-tubulin also accumulates in stable microtubules which can resist to cold treatment and certain drug stimulation [4, 38]. Acetylation and detyrosination of tubulin collectively contribute to microtubule stability, the former being essential for microtubule steadiness [2, 39]. Apart from somatic cells, ac-tubulin was also inspected in oocytes [30]. To date, several studies have indicated the roles of ac-tubulin during oocyte maturation. For example, HDAC3 has been reported to affect the meiotic apparatus assembly by regulation of tubulin acetylation in mouse oocytes [15]. In addition, Esco1, which is involved in sister chromatid cohesion, acetylates α-tubulin to promote proper spindle assembly in meiosis [18]. In our study, we observed that a big proportion of spindle structure was destroyed after Kif18a KD, which correlated with a high tubulin acetylation level in comparison with the control oocytes. Moreover, we showed that Kif18a regulated deacetylase Sirt2 for the tubulin acetylation. Based on the relationship between tubulin acetylation and microtubule stabilization, we speculated that the decrease of deacetylase Sirt2-induced high tubulin acetylation may be the cause for the spindle morphology and chromosome alignment defects in Kif18a-KD oocytes.

Tubulin acetylation normally occurs at lysine 40, and the substitution of lysine (K) with arginine (R) can lead to deacetylation of tubulin.(Chen, [6, 41] Therefore, we generated tubulin K40R mRNA and microinjected it into the Kif18a-KD oocytes, which we could use K40R mRNA to reduce the expression ac-tubulin, which neutralize the increased ac-tubulin caused by Kif18 knock down. This elicited a protective response, since K40R reduced the redundant ac-tubulin caused by Kif18 deficiency. And we showed that the oocytes displaying normal spindle morphology and chromosome alignment after Kif18a KD, which further confirmed the impact of Kif18a on tubulin acetylation in oocytes.

## Conclusions

In summary, our results indicate that Kif18a is essential for spindle organization and chromosome alignment during mouse oocyte maturation via its effects on tubulin acetylation.

## Methods

### Antibodies and chemicals

Mouse monoclonal anti-Kif18a and mouse monoclonal anti-acetylated tubulin antibodies were purchased from Santa Cruz (Santa Cruz, CA, USA). Rabbit polyclonal anti-Kif18a, Sirt2 and Nat10 antibodies was purchased from Proteintech (Proteintech, CHI, USA). Mouse monoclonal anti-α-tubulin-FITC antibodies were from Sigma-Aldrich Corp. (St. Louis, MO, USA). FITC-conjugated and TRITC-conjugated goat anti-rabbit IgG were from Zhongshan Golden Bridge Biotechnology, Co., Ltd. (Beijing, China). All other chemicals and reagents were from Sigma-Aldrich Corp., unless otherwise stated.

### Oocyte harvest and culture

All animal experiments complied with standards formulated by the Animal Care and Use Committee of Nanjing Agriculture University. ICR mice were used in the experiments. All mice were housed under appropriate conditions, including controlled temperature, regular diet and appropriate light. Female mice (3–4 weeks) were used to collect GV oocytes. To obtain GV oocytes, cumulus-enclosed oocytes were obtained by manual rupturing of antral ovarian follicles. Cumulus cells were removed by repeated pipetting. For in vitro maturation, GV oocytes were cultured in M16 (Sigma) medium under mineral oil, at 37 °C, in a 5% CO_2_ atmosphere.

### Nocodazole and Taxol treatment of oocytes

For the nocodazole treatment, oocytes were incubated in medium containing 20 μg/ml nocodazole for 20 min. Then, these oocytes were fixed to undergo further experiments. For the taxol treatment, oocytes were incubated in culture medium containing 10 μM taxol for 40 min. Then, these oocytes were fixed to undergo further experiments.

### Plasmid construct and in vitro transcription

Template RNA was generated from 10 ovaries with an RNA Isolation Kit (Thermo Fisher), and reverse transcription to cDNA was performed by the PrimeScript 1st strand cDNA synthesis kit (Takara, Japan). Vector (pcDNA3.1) cloning with Flag-tubulin substitution mutants (K40R) was conducted by the StarMut site-directed mutagenesis kit (GenStar, Cat#T111-01). mRNA was synthesized from linearized plasmid using the HiScribe T7 high yield RNA synthesis kit (NEB), then capped with m7G(5′)ppp(5′)G (NEB), tailed with a poly(A) polymerase tailing kit (Epicentre), and purified with the RNA clean & concentrator-25 kit (Zymo Research).

### Kif18a siRNA injection

Kif18a siRNA microinjection was used to knock down Kif18a in mouse oocytes. Kif18a siRNA 5′- GCU UCA CUG UCA CCA UUU ATT UAA AUG GUG ACA GUG AAG CTT -3′ (Genepharma, Shanghai, China) was diluted with water to a 75 μM stock solution, and 5–10 pl of siRNA solution was injected into oocytes. Additionally, siRNA 5′-UUC UCC GAA CGU GUC ACG UTT ACG UGA CAC GUU CGG AGA ATT-3′ (5–10 pl) (Genepharma, Shanghai, China) was injected as a control. After injection, the oocytes were cultured in M16 medium containing 5 μM milrinone for 24 h, then washed three times, each for 2 min, in fresh M2 medium. The oocytes were then transferred to fresh M16 medium and cultured for 8 h or 12 h to detect spindle and chromosome organization or to determine their maturation status (polar body extrusion), respectively.

### Confocal microscopy

Oocytes were fixed in 4% paraformaldehyde (in PBS), at room temperature, for 30 min, and then permeabilized with 0.5% Triton X-100 in PBS for 20 min. To reduce non-specific IgG binding, oocytes were incubated with blocking buffer (1% BSA-supplemented PBS), at room temperature, for 1 h. Then, oocytes were incubated with a mouse monoclonal anti-Kif18a antibody (1:50), a mouse monoclonal anti-ac-tubulin antibody (1:100) or an anti-α-tubulin-FITC antibody (1:100), respectively, at 4 °C, overnight. After 3 washes (2 min each) with wash buffer (0.1% Tween 20 and 0.01% Triton X-100 in PBS), oocytes were labeled with an appropriate secondary antibody coupled to FITC-conjugated and TRITC-conjugated goat anti-mouse IgG (1:100), at room temperature, for 1 h. Oocytes were then co-stained with DAPI to examine the chromosomes. Next, samples were mounted on glass slides and observed with a confocal laser-scanning microscope (Zeiss LSM 700 META, Germany).

### Western blot analysis

Approximately 150–180 mouse oocytes were placed in Laemmli sample buffer and heated at 90 °C for 10 min. Proteins were separated by SDS-PAGE, at 150 V, for 90 min, and then electrophoretically transferred to polyvinylidene fluoride (PVDF) membranes (Millipore, Billerica, MA, USA), at 20 V, for 60 min. After transfer, membranes were blocked with TBST containing 5% non-fat milk for 1 h, followed by incubation with a rabbit polyclonal anti-Kif18a (1:200), a mouse monoclonal anti-actin antibody (1:1000) and a mouse monoclonal anti-ac tubulin antibody (1:1000), at 4 °C, overnight. After washing 3 times in TBST (10 min each), membranes were incubated at 37 °C, for 1 h, with HRP-conjugated Pierce Goat anti-Rabbit IgG (1:6000) or HRP-conjugated Pierce Goat anti-mouse IgG (1:1000). Finally, the specific proteins were visualized using a chemiluminescence reagent (Millipore, Billerica, MA).

### Statistical analysis

At least three biological replicates were used for each analysis. Each replicate was performed as an independent experiment, at a different time. Results are presented as mean ± SEM. Statistical comparisons were conducted using analysis of variance (ANOVA), and differences between treatment groups were assessed with the *t* test embedded in Prism5 (GraphPad Software, La Jolla, CA, USA). A p-value of < 0.05 was considered significant.

## Abbreviations

GV: germinal vesicle
GVBD: germinal vesicle breakdown
MI: metaphase I
ATI: anaphase and telophase I
MII: metaphase II
KD: knock down
